# Language-based translation and prediction of surgical navigation steps for endoscopic wayfinding assistance in minimally invasive surgery

**DOI:** 10.1007/s11548-020-02264-2

**Published:** 2020-10-10

**Authors:** Richard Bieck, Katharina Heuermann, Markus Pirlich, Juliane Neumann, Thomas Neumuth

**Affiliations:** 1 Innovation Center Computer Assisted Surgery (ICCAS), Leipzig University, Semmelweisstraße 14, 04103 Leipzig, Germany; 2 Department for Ear-, Nose- and Throat-Surgery, University of Leipzig Medical Center, Leipzig, Germany

**Keywords:** Natural language processing, Endoscopic navigation, Machine translation, Workflow prediction, Deep learning, Attention networks, FESS

## Abstract

**Purpose:**

In the context of aviation and automotive navigation technology, assistance functions are associated with predictive planning and wayfinding tasks. In endoscopic minimally invasive surgery, however, assistance so far relies primarily on image-based localization and classification. We show that navigation workflows can be described and used for the prediction of navigation steps.

**Methods:**

A natural description vocabulary for observable anatomical landmarks in endoscopic images was defined to create 3850 navigation workflow sentences from 22 annotated functional endoscopic sinus surgery (FESS) recordings. Resulting FESS navigation workflows showed an imbalanced data distribution with over-represented landmarks in the ethmoidal sinus. A transformer model was trained to predict navigation sentences in sequence-to-sequence tasks. The training was performed with the Adam optimizer and label smoothing in a leave-one-out cross-validation study. The sentences were generated using an adapted beam search algorithm with exponential decay beam rescoring. The transformer model was compared to a standard encoder-decoder-model, as well as HMM and LSTM baseline models.

**Results:**

The transformer model reached the highest prediction accuracy for navigation steps at 0.53, followed by 0.35 of the LSTM and 0.32 for the standard encoder-decoder-network. With an accuracy of sentence generation of 0.83, the prediction of navigation steps at sentence-level benefits from the additional semantic information. While standard class representation predictions suffer from an imbalanced data distribution, the attention mechanism also considered underrepresented classes reasonably well.

**Conclusion:**

We implemented a natural language-based prediction method for sentence-level navigation steps in endoscopic surgery. The sentence-level prediction method showed a potential that word relations to navigation tasks can be learned and used for predicting future steps. Further studies are needed to investigate the functionality of path prediction. The prediction approach is a first step in the field of visuo-linguistic navigation assistance for endoscopic minimally invasive surgery.

## Introduction

Minimally invasive endoscopic surgery is valued as a standard in surgical practice, because with this method patient's trauma and blood loss are reduced, recovery rates are faster compared to open surgery. These advantages of endoscopy, however, come at the expense of a higher surgical workload for orientation. The increased navigational demand is met with assistance systems that provide information on the in situ position of the endoscope. This is achieved by introducing additional tracking hardware, e.g., infrared or electromagnetic sensors, into the operating room (OR) [1]. Since, the hardware configuration and system usability constraint the surgical workflow, efforts are made to virtualize tracking functions by computational means [1].

New navigation systems employ an image-based approach for the registration of images and detection of objects to identify and use the image content of the endoscopic view for computer-assisted guidance in interventions [2]. Concerning the performance, the applied image processing algorithms are highly automatable and considerably reduce the need for tracking hardware and additional imaging information in the OR [3–5]. The virtualization trend has recently been intensified with deep learning applications that use neural networks to classify the observed anatomy and, thereby, the position of the endoscope [6, 7].

We consider image-based deep learning models for endoscope tracking tasks an essential function of future intelligent navigation systems and aim to extend their applicability to future wayfinding tasks. Compared to automotive and aircraft navigation assistance system [8, 9], a critical function that deep learning approaches in endoscopic applications currently lack is the ability to predict the operator’s future actions and potential pathways. The applications mentioned demonstrate that proactive knowledge of the navigation process improves the responsiveness of an assistance system to the operator’s behavior. In endoscopic procedures, where factors specific to the individual surgeon and patient influence the navigation process, a predictive component is equally important.

This paper, therefore, extends the research on deep learning-based endoscopic tracking by investigating a prediction method for endoscope positions along the navigation process. Our main objective is the ability to estimate future endoscope positions, similar to route predictions in car navigation. In this way, surgical goals and needed instruments could be known beforehand to allow allocation of resources in the OR. The predictive information on the navigation process may also improve the ability to estimate the remaining procedure time. Although the advantages of predicting surgical actions for context detection and resource management have already been demonstrated in the OR [10–13], there are currently no prediction models for surgical activities from the perspective of the endoscopic navigation process. More specifically, a machine-interpretable representation of an endoscopic navigation process is missing that could be facilitated in applications for prediction and image classification. The purpose of this paper is, therefore, threefold:

1. We introduce a machine-interpretable representation of the endoscopic navigation process using workflow annotations based on natural language.
2. We establish a method based on natural language processing to predict endoscope positions at a sentence level and
3. We show that—compared to baseline class level predictors—an attention-based sequence-to-sequence model can predict future endoscope positions with reasonable accuracy at sentence level.

Our approach is tested on recordings of functional endoscopic sinus surgery (FESS) with high anatomical complexity but constrains on endoscopic pathways.

## Related work

Currently, image-based deep learning methods determine the endoscope’s position from the classification of endoscopic images using inherent visual features [6, 7]. When image labeling is provided [14], a topological scene association can be formed. A primary benefit of this step is the simplified view of the anatomical environment since only semantic image content is addressed in the labeling of training data. Similar to [6], our approach uses the description of anatomical landmarks for the labeling of image data to simplify the task of predicting navigation steps to an association of subsequent scenes observed with content of landmarks. However, rather than focusing on spatial properties, we want to use the inherent temporal information between labeled images to extract a representation of states between images that show particular endoscope positions.

The usage of temporal features between endoscopic image has been validated in [13] to estimate procedure durations accurately. Furthermore, in [15], the advantage of combining spatio-temporal features for classification tasks was shown, suggesting that latent temporal information between labeled images exists. Alternatively to conventionally training models directly on image features, our approach is oriented on the prediction of time series using statistical models [12, 16]. We consider the prediction of endoscope positions as a time series classification task on workflow data. In [17], the capabilities of using neural models for time series classification tasks have been shown. However, by focusing on class representations, we lose the semantic information described in workflow activities. The fact that this information can be used for prediction scenarios has been shown in hybrid navigation applications featuring rule-based semantic methods [18, 19].

Our approach, therefore, directly trains a neural language model which represents workflow descriptions of navigation steps to consider both semantic workflows and neural time series classification models. The argument of using natural language processing in computer vision tasks is not new. In autonomous and instructed navigation of robotic agents, a form of language commentary offers a higher-level control of path planning actions, while being visually grounded at the same time [20]. We assume that our research may contribute to a form of visual dialog as a new hybrid navigation approach in the future.

## Methods and materials

Initially, we describe our approach for a machine-interpretable description of an endoscopic navigation process. The vocabulary for the description of endoscope positions from endoscopic images was defined first and then used to annotate a sequence of descriptions for each recorded FESS procedure. In this way, individual workflows of the observed endoscope positions across a procedure were generated. Workflow records were then parsed into class- and sentence-level representations as part of the pre-processing steps for training our prediction models. Subsequently, the training parameters and performance metrics were introduced for prediction tasks of neural language and baseline prediction models. Throughout this paper, we use the term “sentence translation” for sentence-level data in analogy to the term “prediction” for class-level data.

### Process description and representation of endoscopic navigation

**Fig. 1:**
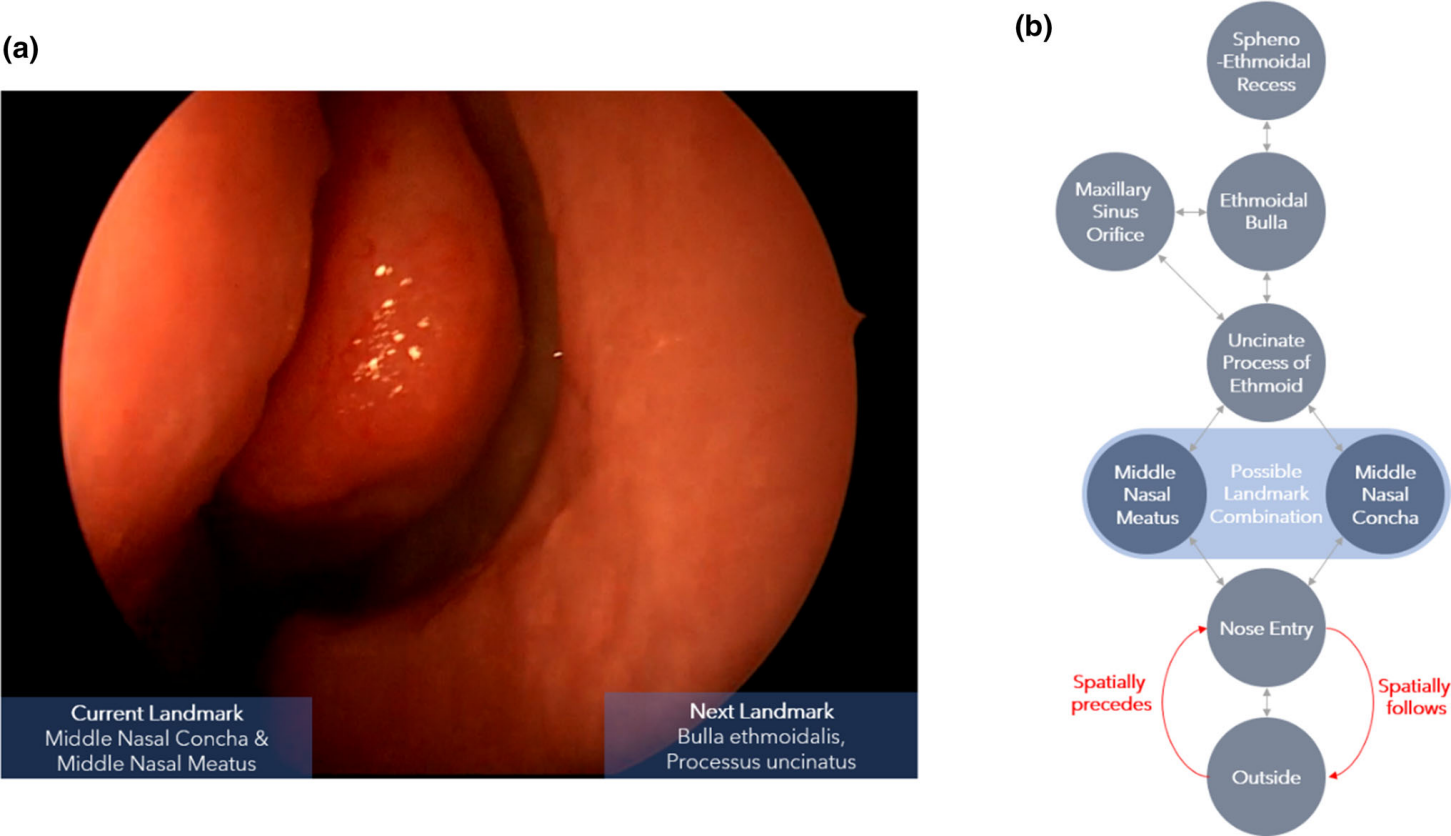
Definitions for the establishment of navigation prediction in functional endoscopic sinus surgery: **b** Example for our natural language processing-based prediction function during a FESS procedure. **a** Possible transitions between endoscopic states that relate to an observed anatomical landmark with examples of an occurring landmark combination (blue) and semantic relations used (red), transitions are bi-directional due to the possible movement between landmarks at any moment in time throughout the procedure

The main objective of our prediction model is the ability to estimate future endoscope positions from a current position (Fig. 1a). From an imaging perspective, the predicted endoscope position could be understood as the most-likely state where a specific anatomical landmark would be visible in an endoscopic view (Fig. 1b). As a requirement for this representation, we established a machine-interpretable description of states for the endoscopic navigation process from research on surgical process analysis in the OR [21]. The strategy assumes that the surgical navigation process is modeled in a bottom-up fashion through the observation of individual workflows during a procedure. Conventionally, surgical workflows would be recorded through observers in the OR. However, our prediction models were to be trained on the label information of images analogous to image-based classifiers and were therefore annotated on recorded endoscopic videos. An annotated endoscopic navigation workflow can thus be understood as a sequence of timed intervals that depict specific endoscopic states. For the descriptions of such states, we established a first description vocabulary based on the FESS use case.

#### FESS navigation workflow

FESS aims to restore the ventilation and drainage function of the paranasal sinuses. The chronically inflammatory tissue is removed, and healthy tissue retained to a large extent. The procedure is surgically demanding due to complex nasal and paranasal structures, as well as adjacent anatomical risk areas such as the *orbita* and the central nervous system. The navigation process through the nasal cavities is characterized by a distinct recurring movement past salient anatomical structures. A preliminary questionnaire with ENT surgeons (*n* = 10, 1–25 years of experience) revealed the importance of information as these guidance landmarks are approached.

**Fig. 2:**
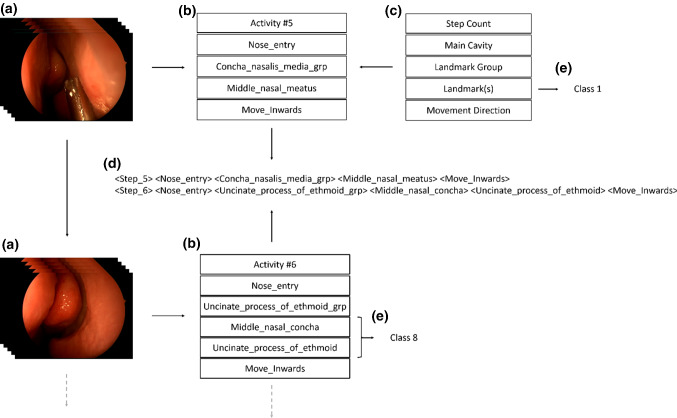
Explanation for the extraction process of annotating endoscopic images with landmark content and parsing into sentence-level descriptions: **a** image frames, where an anatomical landmark was visible in a FESS recording, **b** navigation activity **c** navigation vocabulary, **d** pairwise sentence-level representation of consecutive navigation activities and **e** class representations of consecutive navigation activities

The description of endoscopic states for the FESS use case includes the components: (1) step count, (2) main cavity, (3) landmark group, (4) landmark and (5) direction of movement (Fig. 2c). With (1), we kept track of the actual number of observed landmarks in a FESS. The components (2), (3) and (4) have a structural relation and are included to have different levels of semantic granularity in each state description. The vocabulary for these entities was chosen from the Foundation Model of Anatomy Ontology (FMA)^1^ to have a consistent naming convention. Due to narrow anatomical pathways, landmarks regularly occur in combination and are associated together in landmark groups. This step was required as a basis for the introduction of an ontology of the surgical situation that considers temporal and spatial relations at a landmark level [22]. Through this ontology, component (5) was added to include directional information, two adjacent landmarks. An “inwards” endoscope movement direction occurs, when the currently observed landmark “spatially follows” the one observed before it. Intuitively, a “spatially follows” relation can be seen as the state of a landmark being closer to a defined anatomical center than another one. In response, a ”spatially precedes” condition causes the description “outwards” movement (Fig. 1a red). When two landmarks are spatially equivalent, a “dwell” direction is used. The described vocabulary was then used to annotate FESS recordings (Fig. 2b).

#### Data acquisition and annotation

For 22 patients with similar indications, recordings of a FESS were acquired. Consent was obtained from the patients at a pre-operation discussion, and six different surgeons performed operations. Endoscopic video data were then annotated postoperatively by three surgeons with 2-15 years of surgical experience (mean = 7.2 y) using the SWAN Scientific Workflow Analysis Tool.^2^ In the annotation step, descriptions of the endoscope state were generated using the vocabulary (Fig. 2b and c). Lastly, for each annotated workflow, state sequences were parsed into class- and sentence-level representations. A navigation description parsed into a sentence had between 6 and 10 words. For the training of neural translation models, every two consecutive sentences were paired to create sequence-to-sequence training data (Fig. 2d).

### Sentence-level prediction models

#### Sequence-to-sequence neural networks

For the prediction of navigation steps at sentence-level, neural translation models were chosen that use an encoder-decoder-network structure. In general, the encoder network maps a symbol representation, e.g., a sentence of the source language $x = \left( { x_{0} , \ldots ,x_{n} } \right)$, to a continuous intermediate representation $z = \left( { z_{0} , \ldots ,z_{n} } \right)$. The decoder network then generates an output sentence in the target language $y = \left( { y_{0} , \ldots ,y_{n} } \right)$ one word at a time. These models are autoregressive, using the last output word as input for the generation of the subsequent word to maximize the conditional probability

$$ y = \arg \hbox{max}  p\left( {\hat{y}|x} \right)\quad {\text{with}}\quad p\left( {\hat{y}|x} \right) = \mathop \prod \limits_{i = 0}^{n} p\left( {y_{i} |x} \right) $$

For all possible output words $\hat{y}$. Since, word representations are not distributed independently and they follow the rules for word selection, the network maximizes the likelihood of conditional word probabilities.

**Fig. 3:**
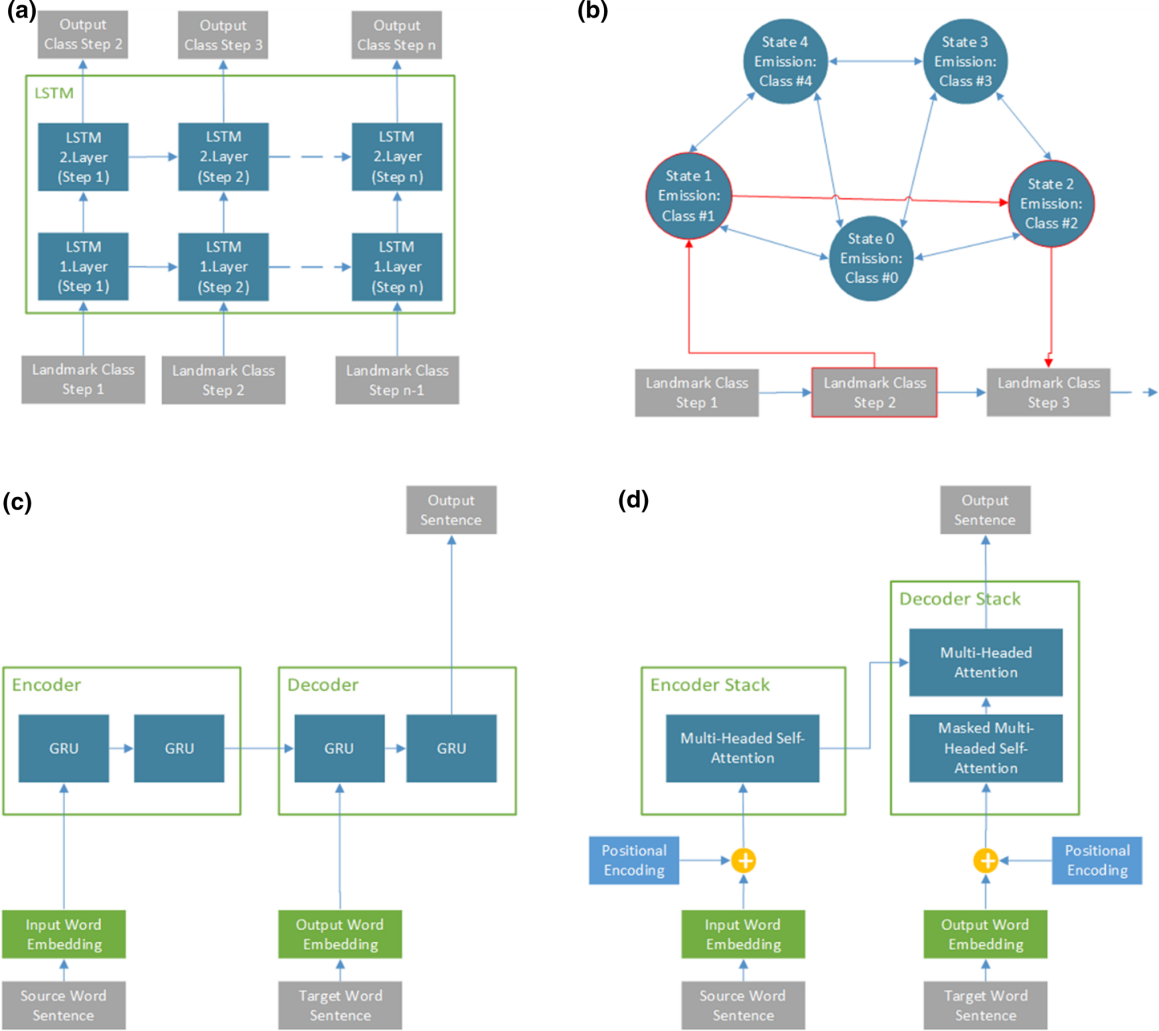
Overview of the prediction models employed: **a** Two-layer long short term memory network, **b** first-order hidden Markov model, **c** standard encoder-decoder-network with gated recurrent units (GRU) and **d** transformer architecture with encoder and decoder stacks and attention blocks

For the sentence-level prediction, we chose a standard encoder-decoder model (S2S) as well as a transformer model (TRF) (Fig. 3a). The S2S model uses two-layered gated-recurrent units (GRU) with 512 neurons in both encoder and decoder. For the TRF, standard parameters from [23] were applied. Both models use word embeddings to encode word relations. We compared both models to investigate if recurrent units or attention-based mechanisms offer significant benefits for sentence prediction (Fig. 3b).

#### Attention-based neural networks

The TRF implementation is adapted from [24] with an additional label smoothing step. A TRF operates using a non-directional approach called attention, where stacks of encoder and decoder layers consider each word simultaneously. We briefly cover the aspects of attention and relate to [25] for the reference architecture. The attention mechanism is defined through the following function:

$$ Attn\left( {Q,K,V} \right) = softmax\left( {\frac{{QK^{T} }}{{\sqrt {d_{K} } }}} \right)V $$

The key-value pairs $$ K,V $$, a positional query $$ Q, $$ and a scaling factor $$ \sqrt {d_{K} } $$ produce a weighted sum output of $$ V $$, using a softmax-function on the dot-product of $$ Q $$ and $$ K. $$ In the TRF network, key-value pairs and query inputs are provided by hidden layer weights. In this way, the TRF aligns its network outputs based on previous weight updates from attention. This mechanism is applied to different sentence sections of the input and output sentences by adding a sinusoid-wave-based positional encoding to sentence embeddings before feeding them to the network.

### Model training and baseline

For the neural translation models training, navigation sentence pairs were tokenized and transformed into standard word embeddings to encode latent semantic information. The training was performed in a leave-one-out cross-validation setup. Left-in datasets were merged and randomly split again into training and validation batches in a 9:1- ratio, resulting in ~ 3300 and 300 sentence pairs, respectively. The source sentences were augmented with random swap and random deletion operations, as described by [26] to improve model generalization further. As baseline models for predicting navigation steps at class-level (Fig. 2e), a first-order hidden Markov model (HMM) with 12 hidden states and expected Gaussian distribution, as well as a 2-layered long-short term memory model (LSTM) with 200 neurons, were chosen (Fig. 3a and b). The S2S and LSTM models converged reasonably fast on this dataset. Both were trained over ten epochs with randomly assembled and shuffled data batches of size $b = 20$ and a cross-entropy loss criterion. The LSTM input sequence length was set to *n* = 6 steps. Batch assembly and shuffling were re-initialized for each epoch to avoid the exhibition of repetitive step-like input behavior. The TRF model converges comparably slower and was trained with the same batch preparation over 40 epochs with the Kullback–Leibler-divergence

$$ \begin{aligned} D_{KL} \left( {P_{\text{Truth}} } \right.||\left. {P_{\text{Pred}} } \right) & = \mathop \sum \limits_{ } P_{\text{Truth}} \left( y \right)\log \left( {\frac{{P_{\text{Truth}} \left( y \right)}}{{P_{\text{Pred}} \left( {\mathop { \, y}\limits^{\prime } } \right)}}} \right) \\ & \equiv H\left( {P_{\text{Truth}} ,P_{\text{Pred}} } \right) - H\left( {P_{\text{Truth}} } \right)  \\ \end{aligned} $$

for discrete probability functions as the loss criterion. $P_{Truth} \left( y \right)$ and $P_{\text{Pred}} \left( {\mathop { \, y}\limits^{\prime } } \right)$ are the ground truth and predicted probability distributions of the labels $y$ and $\mathop { \, y}\limits^{\prime }$ used in the target navigation sentences. The criterion measures the inefficiency of approximating $P_{Truth} \left( y \right)$ with the $P_{\text{Pred}} \left( {\mathop { \, y}\limits^{\prime } } \right)$ and forces the latent variables during training to resemble $P_{\text{Truth}} \left( y \right)$. Additionally, for the longer transformer training process, a label smoothing regularization (smoothing factor = 0.1) was applied to prediction outputs as described by [25]. The regularization step penalizes predicted labels with high confidence, by assigning reduced confidence to the target label scores. The intention is to prevent the model from over-fitting and to improve generalization effects. In all neural network training cases, model weights were updated using an Adam optimizer ($\beta_{1,2} = \left( {0.9,0.98} \right),$ $\varepsilon = 1e - 9$) with warm-up phase and the learning rate

$$ lr = \sqrt {d_{\text{model}} } * \mathop {\hbox{min} }\limits_{ } \left( {n_{\text{step}}^{ - 0.5} ,n_{\text{step}} *n_{\text{warmup}}^{ - 1.5} } \right) $$

$lr$ has linear growth until a warmup step size $n_{\text{warmup}} = 200$ is reached and decreases proportionally to $n_{\text{step}}^{ - 0.5}$ after that. All training and prediction tasks were performed using Pytorch V.1.3.1 [27]. The S2S and TRF model training were finished after 0.5 h and 8.2 h, respectively, with an average processing speed of $\sim 500$ tokens per second. The LSTM model training was finished after 0.4 h. Computations were performed with CUDA 10.1 on an NVIDIA GeForce RTX 2070S graphics card. After training, models exhibited a mean loss of $\bar{l}_{GRU} = 0.282$, $\bar{l}_{TRF } = 0.319$ and $\bar{l}_{LSTM } = 0.262$. The HMM was fitted over 500 iterations using the Baum-Welch-algorithm and reached the convergence threshold of $\varepsilon = 0.01$ in computation time of less than 60 s.

#### Sentence translation and prediction tasks

The trained models were then used to predict navigation steps from the left-out navigation workflows. 3827 sentence pair translations and class predictions were performed. The target navigation sentences were generated one word at a time. For the word candidate search, we implemented an adapted beam search algorithm outlined in [28], and for the context function we used a decaying factor, proposed by [29], to penalize specific beam scores as follows:

$$ s\left( y \right) = \log p\left( {y|x} \right)*\left( {1 - d\left( y \right)} \right)\quad {\text{and}}\quad d\left( y \right) = \left( {1 - e^{{ - \left( {\frac{{r_{y} }}{{r_{y,mean} }}} \right)}} } \right) $$

Here, the score $s\left( y \right)$ of the next word candidate $y$ in the target sentence is calculated as the word candidates’ log-likelihood degraded by a decaying factor $d\left( y \right)$. This factor is defined as an exponential decay function where $r_{y}$ is the current number of subsequent recurrences of the word candidate over the series of navigation sentences and, $r_{y,mean}$ is the mean number of subsequent recurrences of the word candidate overall sentences in the training dataset. The intuition behind this rescoring is that a word candidates’ likelihood is penalized as it is recurring more than the expected mean number of times. This ratio induces a delayed termination of the likelihood of specific word candidates and, thereby, enables other word candidates to be preferred for sentence decoding. Due to our smaller vocabulary size, we used a beam size of 4 instead of typical 16 or 32 beams in more complex text processing tasks.

Predicted and ground truth sentences were then analyzed using similarity metrics BLEU-1 and ROUGE-L (Recall), as proposed in [30], to reflect translation precision and sentence-level structure recall. Based on the scores, we adopted an intermediate $F1$-score:

$$ F_{1,BR} = 2* \frac{BLEU\_1 *ROUGE\_L}{BLEU\_1 + ROUGE\_L} $$

Since both scores are based on n-gram matching, the $F_{1,BR}$ approximates a translation accuracy to produce correct stepwise n-grams. For the sequence-to-sequence models, the predictive power was assessed using a positional-specific accuracy for the classification of the correct words from classification confusions. The word-level Jaccard distance was included to assess dissimilarity. For the baseline model predictions, precision and recall values were calculated.

## Results

### Workflow annotation

A total of 3850 navigation activities was annotated with a mean step number and duration of 167.39 and 2133 s. Between 6 and 16 unique landmark combinations (LMCs) were observed for a mean duration of 9.35 s (LMV). A detailed overview of the workflow properties, as well as the landmark distribution, is provided (Tables 1 and 2). The observed landmarks show an imbalanced distribution toward middle nasal concha and meatus.

**Table 1:** Overview of the results for the annotated surgical navigation workflows. (LMC*—*number of unique landmark combinations in a workflow, mLMV*—*mean landmark visibility duration in a workflow)

Wf	Steps in n	Duration in s	LMC in n	mLMV in s	Wf	Steps in n	Duration in s	LMC in n	mLMV in s
0	81	864	8	13.95	12	238	1896	9	6.97
1	62	876	9	12.35	13	110	1107	9	7.31
2	45	717	6	11.47	14	174	1364	12	5.41
3	19	308	6	10.11	15	453	6775	12	7.33
4	66	600	9	8.44	16	184	2477	10	8.03
5	30	1766	6	13.77	17	200	2233	12	7.25
6	304	3602	9	23.24	18	83	836	9	7.94
7	83	1895	9	37.29	19	208	2439	9	6.90
8	358	5334	11	6.59	20	287	3177	8	7.25
9	248	2996	16	8.38	21	133	1219	8	6.50
10	46	1877	6	4.09	22	123	1282	8	6.71
11	315	3425	14	8.97	$\emptyset$	167.39	2133.26	9.35	10.27

**Table 2:** Overview of the data distribution landmarks observed individually and in combination in absolute values and as observation fractions of the overall dataset

Landmark	Observations, individual		Observations, in combination		Observations, accumulated	
Middle_nasal_concha	885	0.23	1081	0.82	1966	0.51
Middle_nasal_meatus	539	0.14	500	0.13	1039	0.27
Maxillary_sinus_orifice	425	0.11	278	0.07	703	0.18
Out_of_patient	492	0.13	0	0.13	492	0.13
Uncinate_process_of_ethmoid	60	0.01	158	0.04	218	0.06
Ethmoidal_bulla	67	0.01	82	0.02	149	0.04
Spheno_ethmoidal_recess	31	< 0.01	9	< 0.01	40	0.01

### Sentence-level prediction

**Fig. 4:**
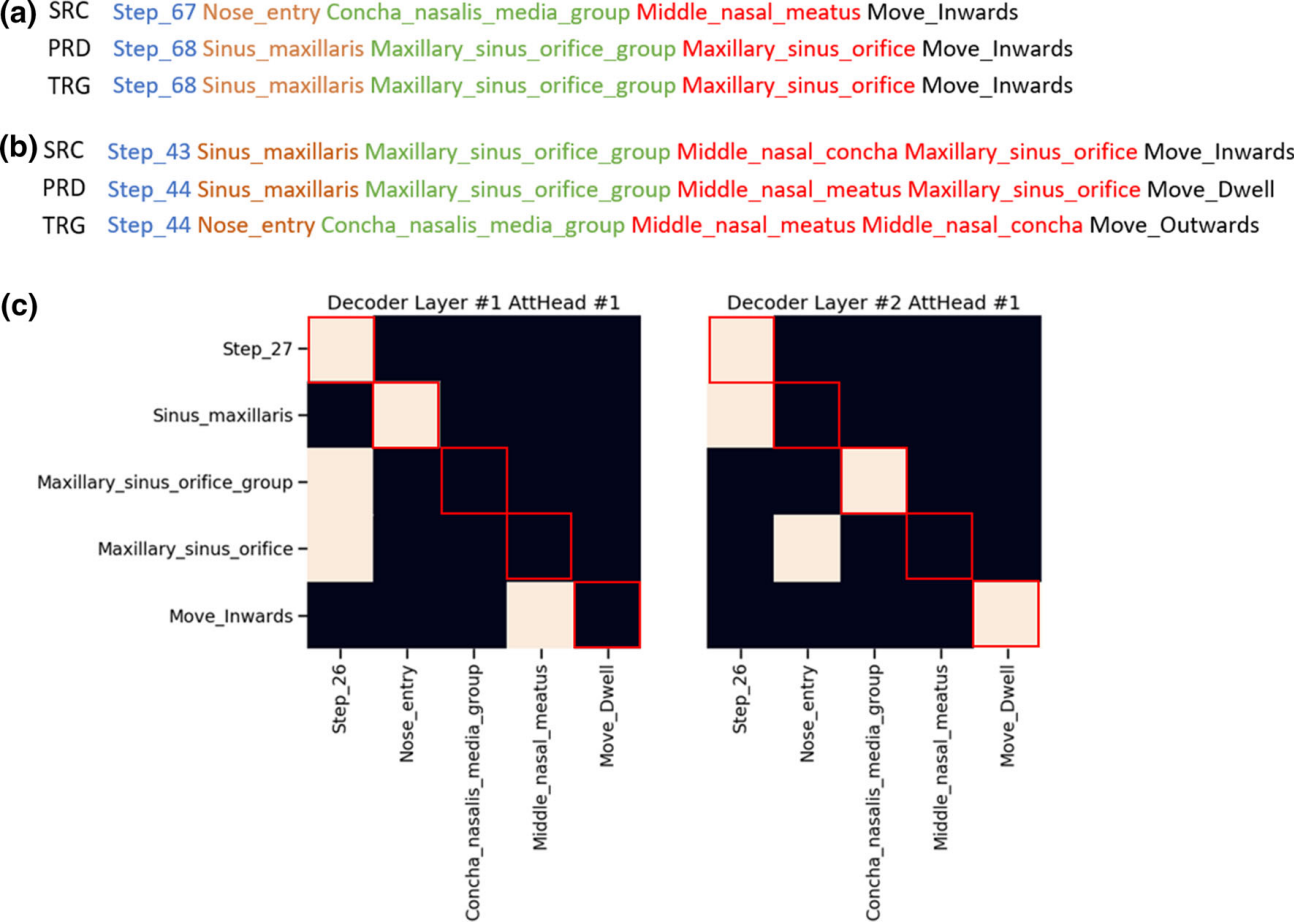
Examples for **a** good and **b** bad sentence translation results generated with the TRF model through decaying beam search decoding (SRC—source sentence, PRD—predicted sentences, TRG—target sentence) as well as **c** examples of the TRF where the training of word relations failed between a current and a future sentence. Red markings show the theoretically ideal weight distribution in the TRF’s decoder when sentence structures are effectively learned

The neural translation models both learned associations between two consecutive navigation sentences. The approximated translation accuracy of 0.75 and 0.83 for the S2S and TRF models indicate that the overall sentence structure was generated correctly (Table 3). In contrast, the accuracy for position-wise predictions was lower, with 0.57 and 0.70 for the S2S and TRF models (Table 4). Accuracy was highest for the step count and lowest for the landmark combinations. Examples for good and bad sentence generations, as well as weight distributions for the TRF attention layer of a low-accuracy sentence generation, are provided (Fig. 4). The TRF displayed lower scores mainly for sentences where word relations between the maxillary sinus and the nose entry area should be predicted.

**Table 3:** Comparison of sentence translation results for the sequence-to-sequence (S2S) and transformer models (TRF). (BL-1—BLEU-1 metric, JD—Jaccard Distance, R-L—ROUGE-L Recall Metric, F1—Approximated Accuracy, higher means better, except for JD, values are averaged)

Model	BL-1	JD	R-L	F1
S2S	0.73	0.29	0.77	**0.75**
Transformer	0.81	0.24	0.87	**0.83**

**Table 4:** Prediction results for the position-specific accuracy of sentence words for the sequence-to-sequence (S2S) and transformer models (TRF**)**. (Pr—precision, Re—recall, higher means better, highest and lowest scores are highlighted)

Sentence Term	TRF		S2S	
	Pr	Re	Pr	Re
Step Count	**0.96**	**0.96**	**0.96**	**0.96**
Sinus	0.74	0.73	0.57	0.51
Landmark Group	0.53	0.73	0.40	0.38
Landmark Combination	0.53	0.60	0.32	0.29
Direction	0.58	0.74	0.55	0.75
Overall	0.67	0.75	0.56	0.57
F1-Score (Accuracy)	**0.70**		**0.57**	

Bold numbers were chosen to highlight important numbers and relevant scores

### Class-level baseline prediction

Comparing the landmark prediction quality of both baseline and translation models, the TRF model performs best with an accuracy of 0.53, followed by the LSTM model for class-based predictions with 0.35 (Table 5). With a precision of 0.94, the HMM scored highest for landmark-specific predictions, but failed to predict four out of the seven other landmarks, entirely. The S2S performed slightly better with 0.32 accuracy. However, the S2S showed an evenly distributed prediction accuracy across all landmarks. Most of the correct predictions were made for the middle nasal meatus, followed by the middle nasal concha and the maxillary sinus orifice.

**Table 5:** Prediction results for the position-specific accuracy of specific landmarks accumulated for individual and in-combination observations using leave-one-out cross-validation. (Pr—precision, Re—recall, higher means better, highest and lowest scores are highlighted)

Landmark	Transformer		LSTM		HMM		S2S	
	Pr	Re	Pr	Re	Pr	Re	Pr	Re
Middle_nasal_concha	0.62	**0.65**	0.67	**0.70**	0.83	0.62	0.42	**0.45**
Middle_nasal_meatus	**0.81**	0.71	**0.69**	0.65	**0.94**	**0.67**	0.36	0.41
Maxillary_sinus_orifice	0.52	0.59	0.36	0.39	0.35	0.58	0.27	0.35
Out_of_patient	0.50	0.43	0.31	0.20	0.00	0.00	0.24	0.34
Uncinate_process_of_ethmoid	0.38	0.34	0.19	0.22	0.00	0.00	0.22	0.20
Ethmoidal_bulla	0.42	0.49	0.22	0.31	0.00	0.00	0.20	0.24
Spheno_ethmoidal_recess	0.50	0.54	0.00	0.00	0.00	0.00	**0.50**	0.23
Overall	0.53	0.53	0.34	0.35	0.30	0.27	0.31	0.32
F1-Score (Accuracy)	**0.53**		**0.35**		**0.28**		**0.32**	

Bold numbers were chosen to highlight important numbers and relevant scores

## Discussion

### Sentence-level prediction

We successfully introduced a workflow representation of the endoscopic navigation process based on the description of anatomical landmarks found in endoscopic images. The comparison of baseline and neural models showed that our proposed natural language-based prediction method at a sentence level performed best for these anatomical landmark sequences. With an accuracy of 0.53, our model outperforms the provided reference models by up to 15%. The attention-based sequence-to-sequence model uses sentence level descriptions of navigation steps and generates sentences with a translation accuracy of 0.83 and a mean word prediction accuracy of 0.70.

Compared to the lower performance of a standard encoder-decoder model, the results suggest that the prediction of sentences benefits from the attention mechanisms of word relations. The achieved landmark and word prediction accuracies are relatable to prediction tasks in the literature, e.g., for procedure state transitions in [18], surgical events by instrument usage in [19] as well as individual phase identification rates in [31]. Furthermore, all of the prediction tasks mentioned above focused on high-level transient processes with minimal or no reoccurring states. Depending on the environmental complexity and type of procedure, a navigation workflow may have multiple recurring activities. Looking at the FESS navigation, sudden changes in movement, e.g., due to exiting the sinus to clean the endoscope lens, were especially hard to learn. Regarding the attention output, bad prediction results always corresponded to missing weight relations between a landmark group and landmark combination words (Fig. 4). This suggests that certain words hold more meaning in our sentence structure than others. The attention-based training of word relations still seems to result in better sentence comprehension than a sequence-to-sequence model using GRU encoders and decoders.

### The representation of navigation processes model restrictions

All trained models show signs of over-fitting (Table 5). Despite the use of an adapted beam search method with decay scoring as well as data augmentation and label smoothing, the TRF model is also mainly constrained by the training data distribution. Noticeably, the LSTM baseline model could map latent temporal properties of navigation workflows even at the class representation level. Both the LSTM and HMM models show a strong bias toward the frequent landmarks. The HMM outputs emissions with more observations in training set using a greedy decoding approach to find the next state. As expected, the HMM switches between the most-likely states and, thus, never reach states associated with other emissions.

Furthermore, the LSTM and HMM models, as well as the GRU-based encoder of the S2S model, fail to capture classes with minimal occurrence. For LSTM and GRU units, this may be caused by the limited sequence length during training and may improve in other training scenarios or with increased training data size. The HMM model may potentially address these classes, but due to the model-fitting aspects mentioned before, the states are never reached. The TRF model possibly alleviates the underrepresentation of specific landmarks by forming unique word relations, e.g., with the step number, the previous central cavity, and the movement direction. This effect is especially critical for navigation scenarios, where specific landmarks are over-represented due to environmental constraints. In the case of the FESS, the landmark distribution is strongly shifted toward the middle nasal area as a central anchor point. This diminishes the quality of prediction of less represented, but highly relevant landmarks. The sequence-to-sequence translation of navigation steps may potentially reduce the effects of imbalanced data through the integration of more semantic content and spatio-temporal relations. Additionally, model architectures with bi-directional information flow, a fixed vocabulary [32] as well as model pre-training and task-specific fine-tuning with customized encoding strategies [33] may offset limitations of the imbalanced annotation data. We point out the approach of [34], where model over-fitting was countered by feeding a sample word from the model-under-training as the next decoded word instead of the ground truth word. In this way, over-fitting toward over-represented landmarks could be further diminished.

### Limitations of prediction

Surgeons who evaluated the predicted steps for the recorded FESS procedures can closely predict navigation steps if no interruptions or irregularities occur during the movement. Still, certain critical events, e.g., a movement direction change of the endoscope and leaving the sinus, are not captured appropriately and, thus, need to be improved to better respond to the surgical process. Overall, prediction results indicate the limitations of the annotation resolution, both temporally and spatially. Here, the addition of a visual-grounding mechanism, e.g., visual attention, could potentially compensate movement recognition restrictions [20]. At the moment, our language-based method assumes an ideal recognition of observable landmarks, which can only assist conventional image-based applications through feedback of predicted steps. The assumed Markov property for the underlying navigation process is a strong assumption and forces sentence relations of short temporal range and reduces the model’s potential for generalization. For further studies, we expect additional assumptions, e.g., Markov chains of higher-order and additional state-dependencies, to improve the robustness of the model and its accuracy further.

## Conclusion

In this work, we validated the feasibility of predicting navigation steps based on the translation of sentences as a verbalized form of annotated navigation workflows. We demonstrated the capabilities of neural machine translation models to generate subsequent navigation sentences and predict navigations steps with an accuracy of 0.83 and 0.53, respectively. Especially the attention mechanism seems to capture word relations in sentence structures even for imbalanced annotation data. However, further studies with an emphasis on a suitable data distribution are needed to thoroughly investigate the performance in predicting navigation workflows. Conventional similarity metrics may pose to be ineffective in assessing model performance from a prediction standpoint.

Our approach for natural language processing of navigation steps differs from current navigation strategies. We consider the implemented sentence-based prediction of navigation steps a form of semantic workflow processing coupled with statistical word representations. Further studies regarding the annotation resolution and vocabulary size, as well as the structure and composition of navigation sentences, are needed to assess the suitability for more complex prediction scenarios. The combination with image-grounded labeling and label-transfer techniques should be consequential, as they are a research focus in the surgical domain. We assume that image-based navigation applications can benefit from the provided predictive information, as the combination is already used in visuo-linguistic navigation tasks in other domains [20].

## Footnotes

1 https://bioportal.bioontology.org/ontologies/FMA.

2 http://www.scientific-analysis.com/.
